# Taoren Honghua Drug Attenuates Atherosclerosis and Plays an Anti-Inflammatory Role in ApoE Knock-Out Mice and RAW264.7 Cells

**DOI:** 10.3389/fphar.2020.01070

**Published:** 2020-07-17

**Authors:** Yiru Wang, Qingyun Jia, Yifan Zhang, Jing Wei, Ping Liu

**Affiliations:** ^1^Department of Cardiology, Longhua Hospital, Shanghai University of Traditional Chinese Medicine, Shanghai, China; ^2^Second Ward of Trauma Surgery Department, Linyi People’s Hospital, Linyi, China; ^3^Department of Traditional Chinese Medicine, Shanghai Xuhui Central Hospital, Shanghai, China

**Keywords:** Taoren Honghua drug, atherosclerosis, inflammation, ApoE knock-out mice, MAPKs, ERK5/STAT3, AKT/NF-κB p65

## Abstract

Taoren Honghua drug is a traditional Chinese medicinal drug used to treat cardiovascular disease. The aim of the study is to investigate the effects of Taoren Honghua drug on inflammation and atherosclerosis in ApoE knock-out mice and RAW264.7 cells. ApoE knock-out mice fed with high fat diet for 8 weeks were randomly divided into five groups and then continued the high fat diet, or plus Taoren Honghua drug at concentrations of 3.63, 1.815, and 0.9075 g/ml, or plus Simvastatin at 2.57 mg/kg. RAW 264.7 cells were intervened with lipopolysaccharide or lipopolysaccharide plus different concentrations of Taoren Honghua drug. Compared to mice only with high fat diet, mice with high fat diet and Taoren Honghua drug showed lower body weight, triglyceride, cholesterol, IL-6 and TNF-α, smaller plaque sizes, less lymph vessel, and T cell contents of lymph nodes, but higher IL-10 level. In RAW264.7 cells, groups with LPS plus Taoren Honghua drug had lower IL-6 and TNF-α, but higher IL-10 than LPS group, as revealed by PCR or ELISA methods. A decrease of total or phosphorylated ERK1/2, JNK, p38, ERK5, STAT3, and AKT were detected, so was the translocation of NF-κB p65 from nuclear to cytoplasm. These results suggested that Taoren Honghua drug could attenuate atherosclerosis and play an anti-inflammatory role *via* MAPKs, ERK5/STAT3, and AKT/NF-κB p65 signaling pathways in ApoE knock-out mice and lipopolysaccharide-induced RAW264.7 cells.

## Introduction

Cardiovascular disease (CVD) is the second leading cause of death in the overall population (Barish et al., 2019) with a prevalence of 48.0% in adults (older than 20 years) according to the data from 2013 to 2016 (Benjamin et al., 2019). The population suffering from CVD is becoming younger (Andersson and Vasan, 2018). Atherosclerosis (AS) is a pathological manifestation in most CVD patients (Conte and Vale, 2018). Inflammatory response runs through the whole AS process (Christodoulidis et al., 2014; Momiyama et al., 2014) and increases the rate of cardiovascular death (Li et al., 2019), providing a new explanation for the pathogenesis of coronary artery disease (CAD) (Tuttolomondo et al., 2012). A clinical trial has verified anti-inflammatory interventions are more precise and personalized for patients with CAD (Libby, 2017). However, the AS guidelines and clinical treatment schemes still aim to lower blood lipids and stabilize plaques (Khan et al., 2015). No targeted treatment for acute and chronic inflammation has been widely applied.

Recently, traditional Chinese medicine (TCM) researchers have been studying the anti-inflammatory effect of TCM herbs on AS (Li et al., 2019). According to TCM, AS is a manifestation of the “blood stasis status”, and therefore should be treated with blood-activating drugs. Previous studies suggested that TCM herbs with blood-activating property could exert anti-inflammatory, anti-platelet aggregation, and anti-ischemia effects on AS (Huang et al., 2011; Wang et al., 2015; Wu et al., 2017). Taoren Honghua drug (THD), based on a classical formula in an ancient book named *Su’an Consilia*, consists of blood-activating herbs. Clinical trials suggested that THD could effectively improve clinical symptoms of CAD caused by AS (Chen, 2011; Li, 2017). However, the underlying mechanism is still unclear.

Hence, we designed this study to explore the attenuating and anti-inflammatory effects of THD on AS in ApoE^−/−^ mice and RAW264.7 cells.

## Methods

### Plant Materials

THD, which contains eleven herbs (**Table 1**), is offered by the Pharmacy of Longhua Hospital Affiliated to Shanghai University of Traditional Chinese Medicine. The herbs were authenticated by a pharmacognosist of Longhua Hospital Affiliated to Shanghai University of Traditional Chinese Medicine, in accordance with standard protocols of the Chinese Pharmacopoeia (Version 2015). All the herbaria are stored in the specific Herbarium Room of Longhua Hospital Affiliated to Shanghai University of Traditional Chinese Medicine. Firstly, all the herbs of the precise weight were soaked in a pot with 400 ml double distilled water (ddH_2_O) for 30 min, Then, the water was boiled for 30 min. After pouring out the liquid in the glassware, another 400 ml of ddH_2_O was added in the same pot and the procedure was repeated. The obtained liquid was mixed and concentrated in a rotary steamer to 100 ml. Finally, 12.25 g lyophilized powder was obtained in a lyophilizer. The powder was resolved in 0.9% saline for oral administration to mice and in Dulbecco’s Modified Eagle Medium (DMEM) high glucose medium supplemented with 10% fetal bovine serum (FBS) for cells intervention. High Performance Liquid Chromatography (HPLC) experiment of THD was conducted (data not shown, **Additional File 1**).

**Table 1:** Components of THD.

Chinese name	Name for publishing*	Amount (g)	Lot No.	Place of origin	Company	Voucher numbers
Tao Ren	*Prunus persica* (L.) Batsch, mature seeds	10	190107	Shanxi, China	Shanghai Hongqiao traditional Chinese medicine decoction pieces Co., Ltd.	*Prunus persica* (L.) Batsch, mature seeds (No.190107-Liu)
Hong Hua	*Carthamus tinctorius* L, dry flower	10	181126	Xinjiang, China	Shanghai Hongqiao traditional Chinese medicine decoction pieces Co., Ltd.	*Carthamus tinctorius L*, dry flower (No. 181126-Liu)
Yan Hu Suo	*Corydalis yanhusuo*, tuber	10	180914	Zhejiang, China	Shanghai Hongqiao traditional Chinese medicine decoction pieces Co., Ltd.	*Corydalis yanhusuo*, tuber (No. 180914-Liu)
Chuan Xiong	*Ligusticum striatum*, rhizome	10	190307	Sichuan, China	Shanghai Yanghetang traditional Chinese medicine decoction pieces Co., Ltd.	*Ligusticum striatum*, rhizome (No. 190307-Liu)
Chi Shao	*Bupleurum sibiricum*, root	10	190110	Inner Mongolia, China	Shanghai Hongqiao traditional Chinese medicine decoction pieces Co., Ltd.	*Bupleurum sibiricu*., root (No. 190110-Liu)
Dan Shen	*Salvia miltiorrhiza* Bunge, root	15	190115	Shandong, China	Shanghai Hongqiao traditional Chinese medicine decoction pieces Co., Ltd.	*Salvia miltiorrhiza* Bunge, root (No. 190115-Liu)
Dang Gui	*Angelica sinensis*, root	12	190108	Gansu, China	Shanghai Hongqiao traditional Chinese medicine decoction pieces Co., Ltd.	*Angelica sinensis*, root (No. 190108-Liu)
Sheng Di	*Rehmannia glutinosa*, root and rhizome	10	190118	Henan, China	Shanghai Hongqiao traditional Chinese medicine decoction pieces Co., Ltd.	*Rehmannia glutinosa*, root and rhizome (No. 190118-Liu)
Qing Pi	Pericarpium Citri Reticulatae Viride, pericarp	10	181001	Zhejiang, China	Shanghai Tongji Tang pharmaceutical Co., Ltd.	Pericarpium Citri Reticulatae Viride, pericarp (No. 181001-Liu)
Xiang Fu	*Cyperus rotundus*, root and rhizome	10	2019010701	Anhui, China	Shanghai Dehua national pharmaceutical products Co., Ltd.	*Cyperus rotundus*, root and rhizome (No. 2019010701-Liu)
Ru Xiang	*Boswellia carteri*, resin	3	18112204	Ethiopia	Shanghai Caitongde Tang decoction pieces Co., Ltd	*Boswellia carteri*, resin (No. 18112204-Liu)

*Latin scientific name + plant part (s).

### Animals

The animal study protocol was approved by the ethics committee of Longhua Hospital Affiliated to Shanghai University of Traditional Chinese Medicine (No. 2019-N002, **Additional File 2**). The ApoE^−/−^ mice were purchased from GemPharmatech Co., Ltd (Nanjing, Jiangsu, http://www.gempharmatech.com) and C57BL/6 mice were from Lingchang BioTech Co., Ltd (Shanghai, China). These mice were bred at the Animal Center of Longhua Hospital Affiliated to Shanghai University of Traditional Chinese Medicine.

Fifty ApoE^−/−^ mice were randomly divided into five groups (10 mice/group): model group (MOD), high THD group (concentration at 3.63 g/ml, TH), medium THD group (concentration at 1.815 g/ml, TM), low THD group (concentration at 0.9075 g/ml, TL), and 2.57 mg/kg Simvastatin group (ST). After 8 weeks’ feeding of high fat diet (HFD), the mice were orally administrated with various concentrations of THD or Simvastatin for 12 weeks. MOD received the same volume of 0.9% saline. Meanwhile, 10 C57BL/6 mice were fed with normal diet without any intervention as the control group (CON). The body weight of the mouse was measured once a week during the experiment.

Simvastatin could decrease the low density lipoprotein cholesterol (LDL-C) level, a key risk factor of CAD, in animal experiments and decrease the levels of triglyceride and total cholesterol in clinical trials. All these are important indexes of arteriosclerotic lesions to evaluate the effects of anti-AS drugs. Therefore, Simvastatin was chosen in the present study as a positive control to evaluate whether THD could play an anti-AS role.

All mice in this study were maintained at the Animal Center of Longhua Hospital Affiliated to Shanghai University of Traditional Chinese Medicine with free access to sterile water and food. At the end of experiments, mice were sacrificed *via* carbon dioxide asphyxiation.

### Cell Culture

To further verify the results of the *in vivo* experiments, we conducted *in vitro* cell experiments. RAW264.7 murine macrophage cell line was purchased from Shanghai Cell Bank Type Culture Collection Committee (Shanghai, China). RAW264.7 cells grown in DMEM high glucose medium supplemented with 10% FBS were maintained at 37°C in a humidified incubator with 5% CO_2_.

RAW264.7 cells (5×10^4^ cells/well) were seeded into 96-well plates and incubated with various concentrations of THD (25, 50, 100, 200, 400, and 800 μg/ml) for 24 h. Cell Counting Kit-8 (Beyotime Biotechnology, China) solution (10 μl/well) was then added to the cells and incubated for 1 h. Then the absorbance of each well was measured under 450 nm to determine cell viability.

Lipopolysaccharide (LPS) was chosen as the inducer because it could enhance inflammation and might promote AS and plaque instability (Sieve et al., 2018). LPS biosynthesis has been proven to up-regulate the atherosclerotic lesion size, plaque area, and the production of IL-4, IL-6, and TNF-α in 16-week-old apolipoprotein E knok out (ApoE^−/−^) mice (Yoshida et al., 2018). Therefore, we believed that LPS-induced RAW 264.7 cells could simulate the inflammation in arteriosclerotic lesions.

For the inhibitors (Sellect, China), RAW264.7 cells were knocked-down (KD) with 100 μg/ml LPS and 10 μM extracellular signal-regulated kinase (ERK) 1/2 inhibitor (PD98059) or c-Jun N-terminal kinase (JNK) inhibitor (SP60012) or p38 inhibitor (SB203580) for 24 h.

### Evaluation of Serum Lipid in the ApoE^−/−^ Mice

Blood samples collected under chloral hydrate anesthesia were kept still for 1 h at room temperature and then centrifuged for 15×min at 18×g. The upper transparent serum of the samples was transferred into new tubes and diluted with ddH_2_O (2:3) for examinations. Total cholesterol (CHOL), triglyceride (TRIG), high density lipoprotein cholesterol (HDL), and low density lipoprotein cholesterol (LDL) levels were analyzed using automatic MODULAR biochemical identification instrument offered by the Clinical Laboratory Department of Longhua Hospital Affiliated to Shanghai University of Traditional Chinese Medicine.

### ELISA for Quantitative Analysis of IL-6, IL-10, and TNF-α

The contents of interleukin (IL)-6, IL-10, and tumor necrosis factor (TNF)-α in blood serum of mice and culture medium of RAW264.7 cells were measured by Enzyme-linked immunosorbent assay (ELISA) kits (Beyotime, China) according to the manufacturer’s instructions.

Mouse blood serum was obtained as described before, and culture medium of cells was derived as follows: RAW264.7 cells (4×10^5^ cells/well) in 96-well plates were treated with various concentrations of THD for 0.5 h, followed by incubation with 100 µg/ml LPS for 23.5 h.

### Oil Red O Staining for Mice Aortic Sinus Lipid-Rich Plaque

The heart was sliced into 10-µm-thick cross-sections to reveal the atrium and aortic sinus. Frozen slices were made as follows: the fresh tissues were fixed in 4% paraformaldehyde at 4 °C for 24 h, then dehydrated in 10%, 20%, and 30% sucrose solutions for 24 h, respectively, and finally embedded with OCT glue. Frozen slices were stained with Oil Red O solution (0.5% in isopropanol, diluted with ddH_2_O in ratio of 3:2) for 1 h at room temperature and counterstained with hematoxylin for 2 min. The Oil Red O stained lipid-rich plaque areas were quantified using ImageJ Software. Data were expressed as the percentage of positive staining in total intimal area.

### Immunofluorescence Staining for Lymphatic Vessels in Heart Atrium and T Cells in Mediastinal Lymph Nodes

Frozen slices of heart atrium and mediastinal lymph nodes were made as mentioned before. On the first day, frozen slices were fixed with 4% paraformaldehyde for 20 min and transparent with 0.1% Triton for 15 min. After being washed for three times with phosphate buffer saline (PBS), the slices were incubated with bovine serum albumin (BSA) solution for 1 h, then with anti-LYVE-1 (for lymphatic vessels in heart atrium) and anti-CD3 (for T cells in mediastinal lymph nodes) (Abcam, UK) at 4°C overnight. On the second day, after washing twice with PBS, the slices were incubated with goat anti-rabbit IgG H&L (Alexa Fluor^®^ 488, CST, USA) at room temperature for 1 h. Then they were washed twice with PBS and mounted after staining with 4’,6-diamindino-2-phenylindole (DAPI). The immunofluorescence staining results were analyzed with ImageJ Software.

### Extraction and Reverse of RNA and Real-Time Quantitative PCR

Gene expressions of IL-6, IL-10, and TNF-α at mRNA level within the mice aorta and cells were tested. RAW264.7 cells were pre-protected with THD for 0.5 h and then stimulated with LPS for 23.5 h.

Total RNA was extracted using RNA Purification Kit (EZBioscience, CN) according to the manufacturer’s instructions. 1 μg RNA was used for reverse transcription to synthesize 20 μl cDNA using PrimeScript RT Reagent Kit (TAKARA, China) according to the manufacturer’s instructions. Then 60 μl ddH_2_O was added to dilute it to 80 μl.

In real-time quantitative PCR (RT-PCR) using TB Green Premix EX Taq with fluorescence quantitative PCR instrument (Applied Biosystems), 4 μl of total RNA was incubated with 1 μl primer (0.1 µg/µl), 10 μl TB Green, 0.4 μl ROXII enzyme, and 4.6 µl ddH_2_O. PCR primers for the IL-6, IL-10, TNF-α, and β-actin were designed using online website PrimerBLAST of NCBI (National Center for Biotechnology Information) and synthesized by Sangon Biotech Co., Ltd. (Shanghai, China). Detailed sequence of primers was listed in **Table 2**.

**Table 2:** List of primers for real-time PCR analysis.

Gene	Oligonucleotide sequence	
β-actin	Forward	5′- ACTGTCGAGTCGCGTCC-3′
	Reverse	5′- CCCACGATGGAGGGGAATAC-3′
IL-6	Forward	5′-GCCTTCTTGGGACTGATGCT-3′
	Reverse	5′-GTGACTCCAGCTTATCTCTTGGT-3′
IL-10	Forward	5′-GTGGAGCAGGTGAAGAGTGA-3′
	Reverse	5′-TCGGAGAGAGGTACAAACGAG-3′
TNF-α	Forward	5′-AGGCACTCCCCCAAAAGATG-3′
	Reverse	5′-TTGAGAAGATGATCTGAGTGTGAG-3′

### Western Blotting

Mouse aortas (mashed manually after frozen with liquid nitrogen) or cells (after washing twice with PBS) with radio immunoprecipitation assay (RIPA) lysis buffer were collected into 1.5 ml microcentrifuge tubes to fully dissociate for 30 min (shaken on a vortex machine completely every 10 min). The whole protein was extracted from the supernatant after centrifugation. The protein contents were measured with the bicinchoninic acid (BCA) protein assay kit (Beyotime, China), separated with 10% sodium dodecyl sulfate polyacrylamide gel electrophoresis for 80 min and transferred onto a polyvinylidene fluoride (PVDF) membrane for 50 min. After being blocked with 2.5% BSA for 1 h, the membrane was incubated in a sealed plastic box with each primary antibody diluted using 2.5% BSA overnight at 4°C. Subsequently, the membrane was washed with Tris-buffered saline with Tween-20 (TBST) and incubated with the appropriate secondary antibody for 1 h. After washing the membrane with TBST five times for 50 min, the signal was visualized using an electrochemiluminescence (ECL) western blot kit (Beyotime, China). The bands were visualized and photographed using the ChemiScope 6000 imaging machine (CLINX, China).

RAW 264.7 cells (6×10^5^ cells/well) with 30 min THD pre-treatment was treated with LPS for 30 min because phosphorylated-proteins levels maxed in 30 min (Wei et al., 2016).

### Statistical Analysis

The results were shown as the mean ± standard deviation (SD). One-way analysis of variant (ANOVA) test was used to determine the statistical significance between groups. Statistical significance was determined at p < 0.05. All experiments were conducted three times or as indicated.

## Results

### THD Attenuates Body Weight and Serum Lipid Profiles in Mice

**Figure 1:**
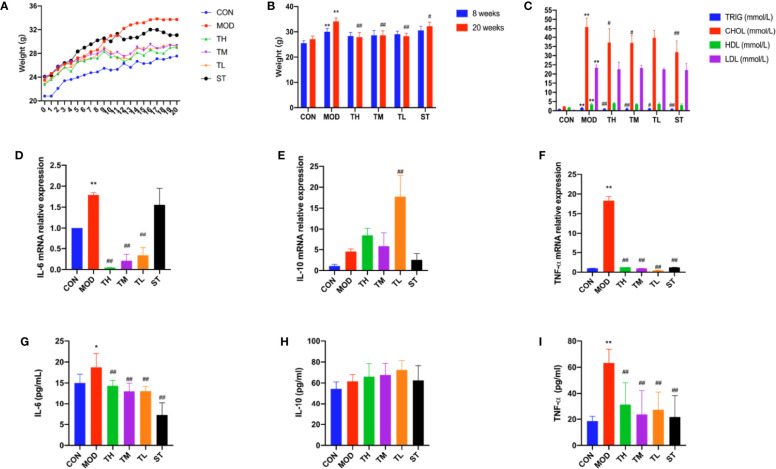
Body weight, serum lipid profiles and inflammatory cytokines in the ApoE^−/−^ mice. Body weight are shown **(A, B)**. −9 to −8 period means one week’s adaption to environment, −8 to 0 period means 8 weeks’ HFD and 0 to 12 period means 12 weeks’ THD or ST intervention. TRIG, CHOL, HLD, and LDL levels of serum were detected **(C)**. Production of IL-6, IL-10, and TNF-α in plasma were measured by ELISA kit **(D–F)**. Aorta gene expression of IL-6, IL-10, and TNF-α were detected by RT-PCR method **(G–I)**. CON means control group, MOD means model group, TH means high THD group, TM means medium THD group, TL means low THD group, ST means Simvastatin group. TRIG, triglyceride; CHOL, total cholesterol; HDL, high density lipoprotein cholesterol; LDL, low density lipoprotein cholesterol. MOD group was compared to CON group and THD or ST group was compared with MOD group. Data are expressed as mean ± SD. *P < 0.05, **P < 0.01 versus CON group; ^#^P < 0.05, ^##^P < 0.01 versus MOD group.

The mice in the MOD, THD, and ST groups had much higher body weights than those in the CON group after 8 weeks (P< 0.01); while after 12 weeks’ intervention of THD or ST, the body weight of mice in the THD and ST groups was significantly lower than those of the MOD group at the 20^th^ week (P< 0.05, P< 0.01, **Figures 1A, B**).

TRIG, CHOL, HDL, and LDL of serum samples were explored. As shown in **Figure 1C**, all the four indexes in MOD group were higher than those in CON group (P< 0.01). Compared to the MOD group, TRIG levels in all the intervention group were lower (P< 0.01, P< 0.05), while CHOL levels in TH, TM, and ST groups were lower (P< 0.01, P< 0.05). However, no significant differences were found in the HDL and LDL levels.

### THD Attenuates Inflammatory Cytokine Expression in Mice

The plasma levels of three typical inflammatory cytokines, IL-6, IL-10, and TNF-α, were detected by ELISA method. As shown in **Figures 1D–I**, the plasma levels of IL-6 and TNF-α in MOD group increased compared with those in CON group (P<0.05, P<0.01), but after 12 weeks’ intervention of THD or ST, the levels of IL-6 and TNF-α were significantly decreased (P<0.01, P<0.01). However, the plasma level of IL-10 expression showed no significant difference among these groups except in TL group as compared to the MOD group (P<0.05). These results were consistent with the changes of mRNA level in mouse aortas as revealed by RT-PCR (P<0.01, P<0.01), except for the IL-6 mRNA level in ST group (no significant change).

### THD Decreased Atherosclerotic Lesion Area in Aortic Sinus of Mice

**Figure 2:**
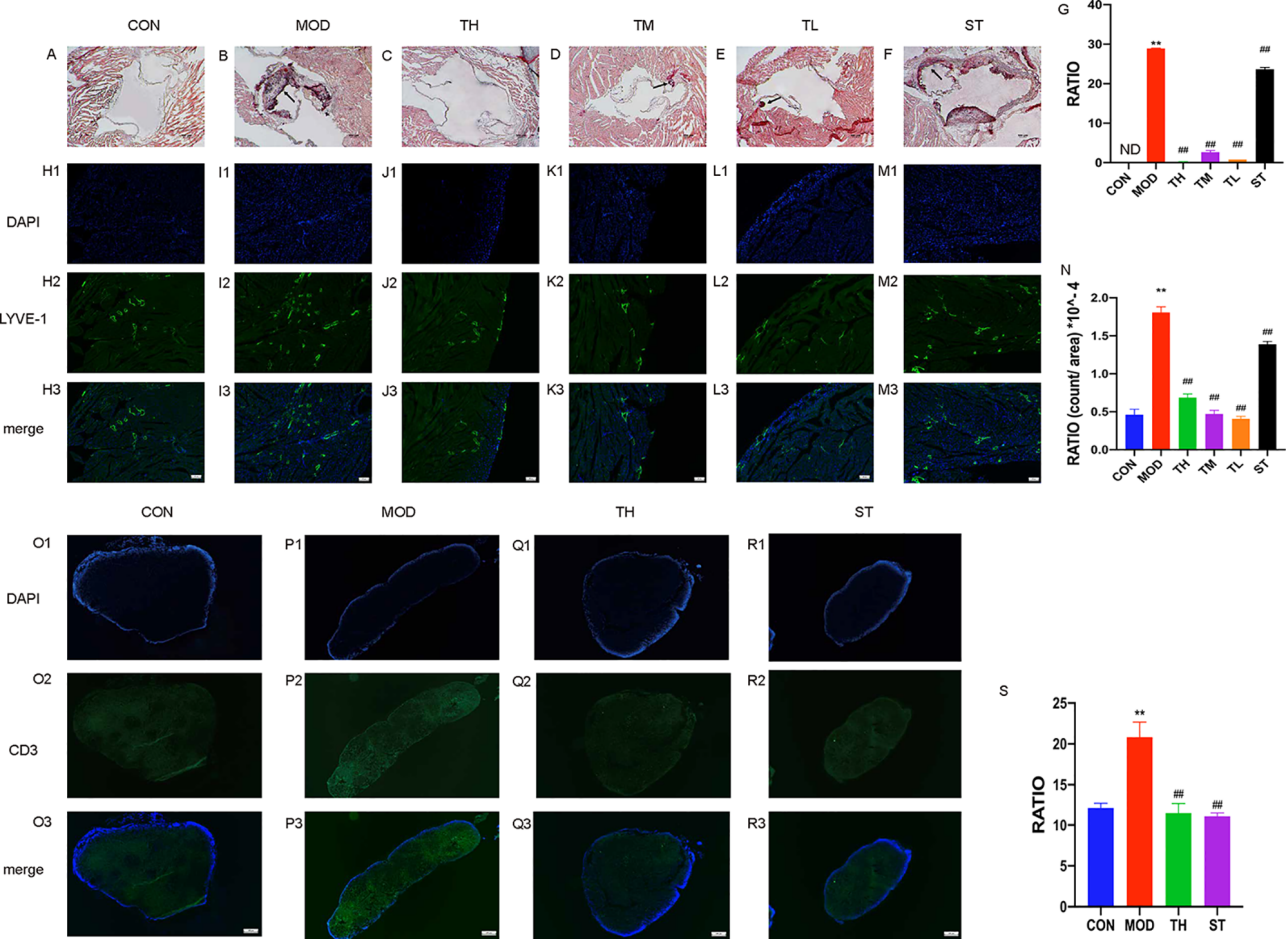
Atherosclerosis of aortic sinus in mice. Oil Red O staining was used to detect the progress of lipid-rich plaques and the representative image of CON group **(A)**, MOD group **(B)**, TH, TM, and TL group **(C–E)**, and ST group **(F)** are shown. Immunofluorescence staining method was used to show the lymphatic vessels of mice heart atrium **(H1–M3)** and contents of T cells in mediastinal lymph nodes **(O1–R3)**. The areas of lesion **(G)**, the numbers of lymphatic vessels **(N)** and T cell **(S)** were calculated respectively by ImageJ analysis software. CON means control group, MOD means model group, TH means high THD group, TM means medium THD group, TL means low THD group, ST means Simvastatin group. MOD group was compared to CON group and THD or ST group was compared with MOD group. Data are expressed as mean ± SD. **P < 0.01 versus CON group; ^##^P < 0.01 versus MOD group.

The progress of lipid-rich plaques was assessed by Oil Red O staining. As shown in **Figures 2A–F**, lipid-rich plaques were clearly observed in ApoE^−/−^ AS model mice, but not detected in mice of the CON group (P< 0.01). In contrast, a significant reduction of plaque size was detected in the THD-treated and ST-treated groups (P< 0.01) compared to MOD group (**Figure 2G**).

### THD Reduced Density of Lymphatic Vessels in Heart Atrium and Number of T Cells

To observe the effects of THD on lymphatic vessel proliferation, LYVE-1 expression of epicardium in heart atrium was examined by immunofluorescence staining. As shown in **Figures 2H1–M3**, the density of lymphatic vessels increased, and their shapes were more discontinuous compared with CON group (P< 0.01). However, the densities of lymphatic vessels in THD and ST groups were significantly lower than that in MOD group (P< 0.01) (**Figure 2N**).

As CD3 was a main marker of T cells, we evaluated the contents of T cells in mediastinal lymph nodes through immunofluorescence staining. As shown in **Figures 2O1–R3**, the T cell content of MOD group was higher than that of CON group, and after TH and ST intervention, the T cell content decreased (**Figure 2S**).

### Optimal THD Concentration for RAW264.7 Cells

Since 5 µg/ml THD did not result in any significant cytotoxicity in LPS-induced RAW264.7 cells, subsequent experiments were conducted with concentrations at 5, 2.5, and 1.25 µg/ml.

### THD Attenuates Inflammatory Cytokine Levels in RAW264.7 Cells

**Figure 3:**
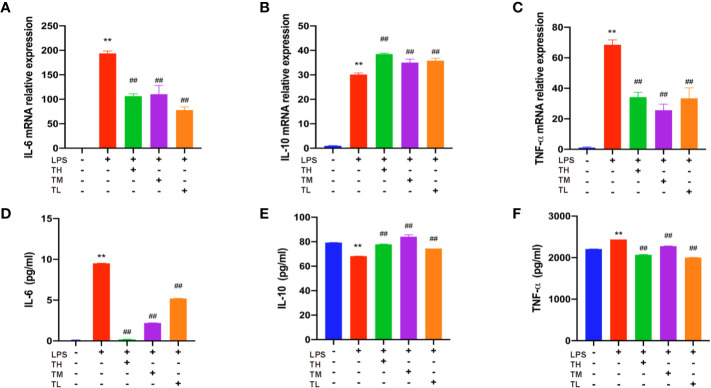
Inflammatory cytokine levels in RAW264.7 cells. Gene expression of IL-6, IL-10, and TNF-α was measured by RT-PCR method **(A–C)** and production of these three cytokines were explored by ELISA kits **(D–F)**. TH means THD at 5 µg/ml concentration, TM means THD at 2.5 µg/ml concentration, TL means THD at 1.25 µg/ml concentration. Data are expressed as mean ± SD. **P < 0.01 versus group without LPS and THD; ^##^P < 0.01 versus group with LPS only.

Expressions of IL-6, IL-10, and TNF-α in RAW264.7 cell supernatant were detected in order to further confirm the results of the above observation. RAW264.7 cells were pretreated with THD for 0.5 h and then stimulated with LPS for 23.5 h. As shown in **Figures 3A–F**, the expression levels of IL-6 and TNF-α increased, while the IL-10 expression decreased in the LPS group (P< 0.01). After THD intervention, IL-6 and TNF-α fell while IL-10 rose in ELISA kit detection (P< 0.01). These results were consistent with the changes in mRNA level as revealed by RT-PCR (P< 0.01).

### THD Inhibited MAPKs-Related Protein Expression Levels in Mice and RAW264.7 Cells

**Figure 4:**
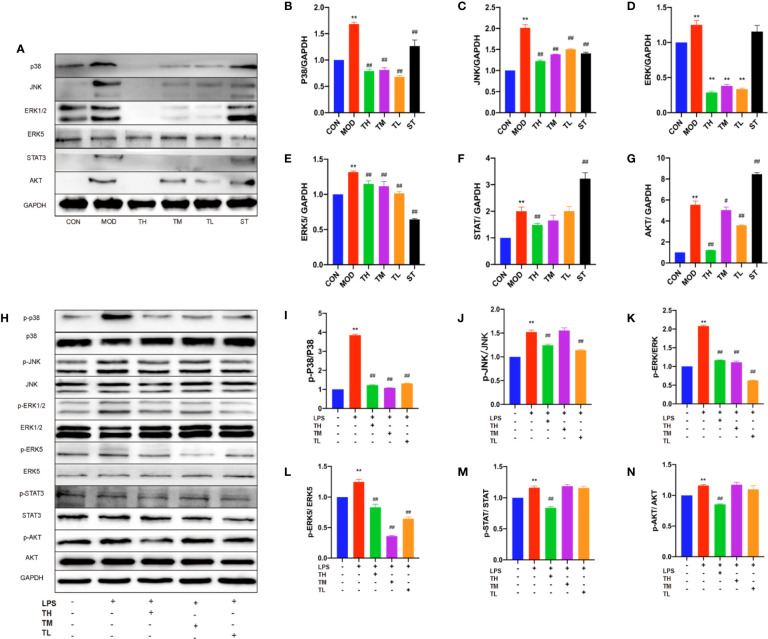
Effects of THD on different signaling pathways in mice and LPS-induced RAW264.7 cells. Protein was extracted from mice aorta. Then p38, JNK, ERK1/2, ERK5, STAT3, and AKT were tested by western blotting assay **(A)**. Protein was extracted from RAW264.7 cells. The total and phosphorylated levels of p38, JNK, ERK1/2, ERK5, STAT3, and AKT were determined by western blotting assay **(H)**. The quantitative results were depicted **(B–G, I–N)**. In **Figures 4A–G**, CON means control group, MOD means model group, TH means high THD group, TM means medium THD group, TL means low THD group, ST means Simvastatin group. Data are expressed as mean ± SD. ** P < 0.01 versus CON group; ^#^P < 0.05, ^##^P < 0.01 versus MOD group. In the **Figures 4H–N**, TH means THD at 5 µg/ml concentration, TM means THD at 2.5 µg/ml concentration, TL means THD at 1.25 µg/ml concentration. Data are expressed as mean ± SD. **P < 0.01 versus group without LPS and THD; ^##^P < 0.01 versus group with LPS only.

In order to testify whether the increased inflammatory cytokines, lymphatic vessels and lymph nodes were related to MAPKs, total p38, JNK, and ERK1/2 were examined by WB assays (**Figure 4A**). The expression of MAPKs in MOD group was higher than that in CON group (P< 0.01), lower than those in THD and ST groups (P< 0.01) except for ERK1/2 expression in ST group (**Figures 4B–D**).

To further testify the above results, 5, 2.5, and 1.25 μg/ml THD-treated LPS-induced cells and phosphorylation of p38, JNK, and ERK1/2 were examined (**Figure 4H**), which showed a significant reduction at the phosphorylation levels of these kinases except for p-JNK/JNK in 2.5 μg/ml THD group (**Figures 4I–K**).

### THD Suppressed ERK5/STAT3 Activation

ERK5/signal transducer and activator of transcription (STAT) 3 pathway was also investigated. As shown in **Figure 4A**, ERK5 of mouse aorta in THD and ST groups decreased (P< 0.01), while STAT3 was reduced in TH group (P< 0.01) but rose in ST group (P< 0.01) (**Figures 4E, F**).

In RAW 264.7 cells (**Figure 4H**), p-ERK5/ERK5 was lower in all the THD groups compared to MOD group (P< 0.01), but p-STAT3/STAT3 was decreased only in TH group (P< 0.01) (**Figures 4L, M**).

### THD Suppressed AKT Activation and NF-κB p65 Translocation

We also investigated the effects of THD on AKT/NF-κB p65 nuclear translocation—the key regulator of AS. As shown in **Figure 4A**, AKT of mouse aorta in THD groups was decreased compared to that in MOD group (P< 0.05, P< 0.01) (**Figure 4G**). Besides, p-AKT/total AKT of RAW 264.7 cells was decreased in TH group compared to that in MOD group (P< 0.01) (**Figures 4H, N**).

**Figure 5:**
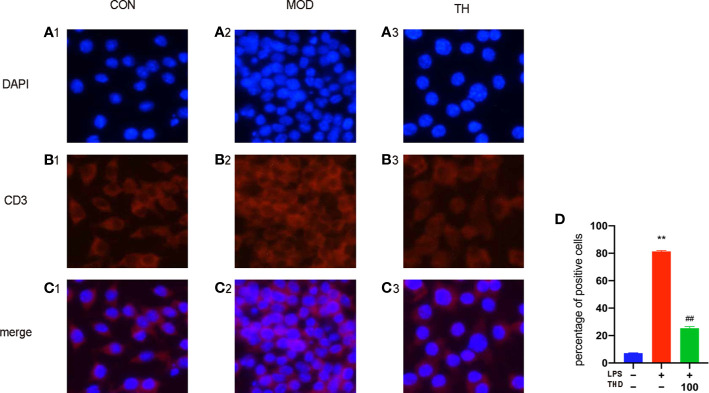
NF-κB p65 signaling pathway in LPS-induced RAW264.7 cells. Immunofluorescence staining method was used to show the NF-κB p65 translocation (**A**1–**C**3) The ratios of positive cells were calculated by ImageJ analysis software **(D)**. CON means control group, MOD means model group, TH means high THD group, ST means Simvastatin group. MOD group was compared to CON group and THD or ST group was compared with MOD group. Data are expressed as mean ± SD. **P < 0.01 versus CON group; ^##^P < 0.01 versus MOD group.

Moreover, NF-κB p65 of RAW264.7 cells was decreased in the cytoplasm and increased in the nucleus in the LPS-stimulated group compared to CON group. After THD (5 μg/ml) intervention, NF-κB p65 levels decreased in the nucleus, suggesting that THD significantly decreased AKT activation and NF-kB p65 translocation into nuclei (**Figures 5A1–D**).

### THD Decreased Inflammatory Cytokine Levels in MAPKs-KD RAW264.7 Cells

**Figure 6:**
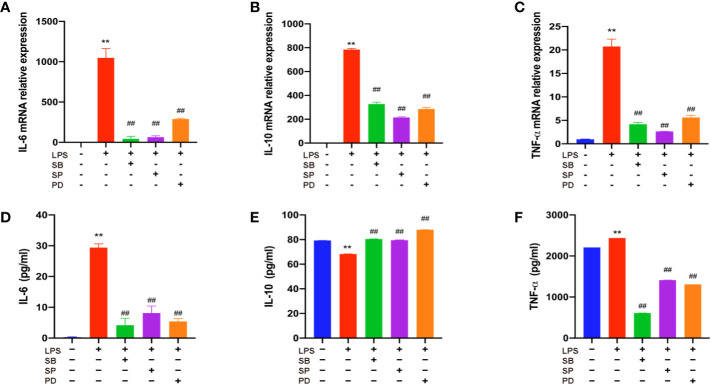
Further investigation of effect of THD on MAPKs signaling pathway. Gene expression of IL-6, IL-10, and TNF-α was detected by RT-PCR **(A–C)**. IL-6, IL-10, and TNF-α production in the supernatant was examined through ELISA kits **(D–F)**. SB means p38 inhibitor, SP means JNK inhibitor, PD means ERK1/2 inhibitor. Data are expressed as mean ± SD. **P < 0.01 versus group without LPS and inhibitors; ^##^P < 0.01 versus group with LPS only.

To further verify the role of MAPKs in the anti-inflammatory function of THD, three specific inhibitors (SB203580, a p38 inhibitor; SP60012, a JNK inhibitor; PD98059, an ERK1/2 inhibitor) were employed (**Figures 6A–F**). These inhibitors significantly blocked the production of IL-6, IL-10, and TNF-α as shown in ELISA analysis (P< 0.01). Additionally, gene expressions of IL-6, IL-10, and TNF-α were dramatically reduced by inhibitors compared to the LPS group (P< 0.05, P< 0.01).

## Discussion

Chronic inflammation worsens the condition by increasing cytokines (Patil et al., 2019). As CAD or AS is associated with inflammation, anti-inflammation is an effective therapeutic strategy to prevent disease progression (Gaudino and Crea, 2019).

The main finding of this study is the role of THD in reducing atherosclerotic area in aortic sinus by triggering anti-inflammatory response. The anti-inflammatory effect of THD found in this study is consistent with the previous research (Sen, 2016). Clinical studies also suggested that THD could improve blood stasis status and clinical symptoms of CAD patients (Weibin and Zheng, 2011; Junhui, 2017). In this research, we also found THD could decrease TRIG and CHOL levels, as well as body weight. Moreover, THD intervention decreased IL-6 and TNF-α and increased IL-10 in mice and cells. In our study, the reason why the IL-10 mRNA level and protein level did not match could be explained as follows: the translation of mRNA into protein is very complex. Non-coding RNAs might decrease the production of protein; even though the protein was produced, posttranslational modification could have an effect on the function and detection of protein; the samples used to detect mRNA or protein levels were different. In **Figure 1H**, the samples to detect protein production were blood serum of mice, while in **Figure 1E**, the samples to explore mRNA level were total RNA extracted from aorta of mice. In **Figures 3E** and **6E**, the samples used to detect protein production were RAW264.7 cell supernate, while in **Figures 3B** and **6B** the samples to explore mRNA levels were total RNA extracted from RAW 264.7 cells. We considered that IL-10 was a secreting protein, and thus the expression of IL-10 in cell supernate was much higher than that in cells.

According to previous papers, proliferation of lymphatic vessels in the heart and plaques was found in CAD and progressing of AS (Kholova et al., 2011; Telinius and Hjortdal Ve Dmsc, 2019), which could be a new treatment target (Vuorio et al., 2017). Additionally, T cells significantly increased in CAD patient’s peripheral blood and epicardial adipose tissues (Hwang et al., 2016; Mraz et al., 2019). Our results are in accordance with these reports: contents of lymphatic vessels in mouse heart atrium and T cells in mediastinal lymph nodes increased in MOD group. By contrast, THD decreased both lymphatic vessels and T cells.

Recently, studies have suggested that the attenuation of inflammation and AS is strongly related to MAPKs signaling pathway (Jin et al., 2010; Olejarz et al., 2014). To explore the anti-inflammatory mechanism of THD, we detected the total and phosphorylated p38, JNK, and ERK1/2 expression in mice and cells. As shown by the results, THD intervention decreased total MAPKs of mice and phospho-MAPKs of cells. The inhibitors testified that THD could achieve its anti-inflammatory function *via* MAPKs signaling pathway.

ERK5 is a potential therapeutic target in systematic inflammation because of it may restrain the possible side effects (Wilhelmsen et al., 2015). Moreover, Stat3 could control T cell differentiation (Yuan et al., 2019) and promote ERK5 upregulation (Liu et al., 2017). Thus, we investigated total and activated ERK5 and STAT3. Our results revealed that THD could exert the anti-inflammatory effect through ERK5/STAT3 pathway.

AKT plays a role in inflammation-associated diseases (Yan et al., 2018) and NF-κB p65 is a treatment target to control inflammation (Zhou et al., 2014; Zhang et al., 2019). Activated NF-κB can be translocated into nucleus, then the phosphorylated NF-κB becomes the transcription factors of inflammatory genes (Viatour et al., 2005). Several recent studies have highlighted the noteworthy cross-talk between the AKT and MAPK pathways (Enayat et al., 2013). Our data also indicated THD suppressed the expression of pro-inflammatory cytokines through inhibiting AKT/NF-κB p65 activity.

Compared to MOD group, we found that ST could effectively decrease the body weight, triglyceride, total cholesterol, IL-6, TNF-α, plague area, numbers of heart lymphatic vessels, and T cells in lymph nodes through p38/JNK/ERK5. Simvastatin is commonly recognized to have pleiotropic effects including anti-inflammatory effect in mice, which is consistent with the results of IL-6 and TNF-α in our mice experiments. However, Simvastatin seemed less effective compared to the THD groups. According to the HPLC results, we believe that it was the synergism of several compounds in THD, such as the amygdalin, Salvianolic acid B, and Tanshinone, that enhanced the anti-inflammatory effect.

Lots of available theories in TCM address the use of traditional plant-based medicine in CAD treatment, yet few of them has been explored. Thus, the prospect of discovering new anti-inflammatory medications is promising.

In conclusion, the primary mechanism of THD as an anti-inflammatory medicine was explored. THD was verified to suppress MAPKs, ERK5/STAT3, and AKT/NF-κB p65 signaling pathway, leading to a decreased production of pro-inflammatory cytokines in ApoE^−/−^ mice and LPS-stimulated RAW264.7 cells. Therefore, we believe that THD plays a strong anti-inflammatory role and is worth further investigation.

## Data Availability Statement

The raw data supporting the conclusions of this article will be made available by the authors, without undue reservation, to any qualified researcher.

## Ethics Statement

The animal study was reviewed and approved by ethics committee of Longhua Hospital Affiliated to Shanghai University of Traditional Chinese Medicine.

## Author Contributions

PL conceived and designed the experiments. YW and YZ performed the experiments. JW and QJ analyzed the results. YW was a major contributor in writing the manuscript. PL reviewed and edited the final manuscript. All authors contributed to the article and approved the submitted version.

## Funding

This work was supported by the 2018-2020 Three-year Action Plan for Traditional Chinese Medicine Further Development in Shanghai to PL [grant numbers ZY(2018-2020)-CCCX-2002-04]; the National Natural Science Foundation to PL (grant numbers 81873117); the Shanghai Science and Technology Commission to PL (grant numbers 18401900200); and the Pudong New Area Health and Family Planning Commission to PL (grant numbers PW2018D-11).

## Conflict of Interest

The authors declare that the research was conducted in the absence of any commercial or financial relationships that could be construed as a potential conflict of interest.

## Abbreviations

AS, atherosclerosis; ANOVA, analysis of variant; BSA, bovine serum albumin; CAD, coronary artery disease; CHOL, total cholesterol; CON, control group; CVD, cardiovascular disease; DAPI, 4’,6-diamindino-2-phenylindole; ddH_2_O, double distilled water; DMEM, Dulbecco’s Modified Eagle Medium; ECL, electrochemiluminescence; ELISA, enzyme-linked immunosorbent assay; ERK, extracellular signal-regulated kinase; HDL, high density lipoprotein cholesterol; FBS, fetal bovine serum; HFD, high fat diet; HPLC, High Performance Liquid Chromatography; IL, interleukine; KD, knocked-down; LDL, low density lipoprotein cholesterol; LPS, lipopolysaccharide; MAPKs, mitogen-activated protein kinases; MOD, model group; PBS, phosphate buffer saline; PVDF, polyvinylidene fluoride; NCBI, National Center for Biotechnology Information; NF-κB, nuclear factor-kappa B; RIPA, radio immunoprecipitation assay; RT-PCR, Real-time quantitative PCR; SD, standard deviation; ST, Simvastatin group; STAT, signal transducer and activator of transcription; TBST, Tris-buffered saline with Tween-20; TCM, Traditional Chinese Medicine; TH, high Taoren Honghua drug group; THD, Taoren Honghua drug; TL, low Taoren Honghua drug group; TM, medium Honghua Drug group; TNF, tumor necrosis factor; TRIG, triglyceride.

## References

[B1] Andersson C. Vasan R. S. (2018). Epidemiology of cardiovascular disease in young individuals. Nat. Rev. Cardiol. 15, 230–240. 10.1038/nrcardio.2017.154 29022571

[B2] Barish R. Lynce F. Unger K. Barac A. (2019). Management of Cardiovascular Disease in Women With Breast Cancer. Circulation 139, 1110–1120. 10.1161/CIRCULATIONAHA.118.039371 30779651

[B3] Benjamin E. J. Muntner P. Alonso A. Bittencourt M. S. Callaway C. W. Carson A. P. (2019). Heart Disease and Stroke Statistics-2019 Update: A Report From the American Heart Association. Circulation 139, e56–e528. 10.1161/CIR.0000000000000659 30700139

[B4] Chen W. Y. (2011). Clinical Analysis of 100 Cases of Coronary Heart Disease Treated by Taoren Honghua. Health Required 6, 23.

[B5] Christodoulidis G. Vittorio T. J. Fudim M. Lerakis S. Kosmas C. E. (2014). Inflammation in coronary artery disease. Cardiol. Rev. 22, 279–288. 10.1097/CRD.0000000000000006 24441047

[B6] Conte S. M. Vale P. R. (2018). Peripheral Arterial Disease. Heart Lung Circ. 27, 427–432. 10.1016/j.hlc.2017.10.014 29150158

[B7] Enayat S. Ceyhan M. S. Basaran A. A. Gursel M. Banerjee S. (2013). Anticarcinogenic effects of the ethanolic extract of Salix aegyptiaca in colon cancer cells: involvement of Akt/PKB and MAPK pathways. Nutr. Cancer 65, 1045–1058. 10.1080/01635581.2013.850966 24168160

[B8] Gaudino M. Crea F. (2019). Inflammation in coronary artery disease: Which biomarker and which treatment? Eur. J. Prev. Cardiol. 26, 869–871. 10.1177/2047487319829307 30813816

[B9] Huang Y. Yin H. J. Ma X. J. Wang J. S. Liu Q. Wu C. F. (2011). Correlation between Fc gamma R III a and aortic atherosclerotic plaque destabilization in ApoE knockout mice and intervention effects of effective components of chuanxiong rhizome and red peony root. Chin. J. Integr. Med. 17, 355–360. 10.1007/s11655-011-0726-y 21611899

[B10] Hwang Y. Yu H. T. Kim D. H. Jang J. Kim H. Y. Kang I. (2016). Expansion of CD8(+) T cells lacking the IL-6 receptor alpha chain in patients with coronary artery diseases (CAD). Atherosclerosis 249, 44–51. 10.1016/j.atherosclerosis.2016.03.038 27062409

[B11] Junhui L. (2017). Taoren Honghuajian Capsule Combined with Modified Tongmai Dingtong Decoction for Coronary Heart Disease Angina Pectoris. Tradit. Chin. Med. Res. 30, 13–15.

[B12] Jin M. Suh S. J. Yang J. H. Lu Y. Kim S. J. Kwon S. (2010). Anti-inflammatory activity of bark of Dioscorea batatas DECNE through the inhibition of iNOS and COX-2 expressions in RAW264.7 cells via NF-kappaB and ERK1/2 inactivation. Food Chem. Toxicol. 48, 3073–3079. 10.1016/j.fct.2010.07.048 20691245

[B13] Khan R. Spagnoli V. Tardif J. C. L’allier P. L. (2015). Novel anti-inflammatory therapies for the treatment of atherosclerosis. Atherosclerosis 240, 497–509. 10.1016/j.atherosclerosis.2015.04.783 25917947

[B14] Kholova I. Dragneva G. Cermakova P. Laidinen S. Kaskenpaa N. Hazes T. (2011). Lymphatic vasculature is increased in heart valves, ischaemic and inflamed hearts and in cholesterol-rich and calcified atherosclerotic lesions. Eur. J. Clin. Invest. 41, 487–497. 10.1111/j.1365-2362.2010.02431.x 21128936

[B15] Li S. M. Li J. G. Xu H. (2019). A New Perspective for Chinese Medicine Intervention for Coronary Artery Disease: Targeting Inflammation. Chin. J. Integr. Med. 25, 3–8. 10.1007/s11655-018-2995-1 30132247

[B16] Li J. H. (2017). Taoren Honghua Drug Combined with Modified Tongmai Dingtong Decoction for 76 Cases of Angina Pectoris Due to Coronary Heart Disease. Tradit. Chin. Med. Res. 30, 13–15. 10.1007/s11655-018-2995-1

[B17] Libby P. (2017). Interleukin-1 Beta as a Target for Atherosclerosis Therapy: Biological Basis of CANTOS and Beyond. J. Am. Coll. Cardiol. 70, 2278–2289. 10.1016/j.jacc.2017.09.028 29073957 PMC5687846

[B18] Liu F. Zhang H. Song H. (2017). Upregulation of MEK5 by Stat3 promotes breast cancer cell invasion and metastasis. Oncol. Rep. 37, 83–90. 10.3892/or.2016.5256 27878304

[B19] Momiyama Y. Adachi H. Fairweather D. Ishizaka N. Saita E. (2014). Inflammation, Atherosclerosis and Coronary Artery Disease. Clin. Med. Insights Cardiol. 8, 67–70. 10.4137/CMC.S39423 PMC5003124 27594791

[B20] Mraz M. Cinkajzlova A. Klouckova J. Lacinova Z. Kratochvilova H. Lips M. (2019). Coronary Artery Disease Is Associated with an Increased Amount of T Lymphocytes in Human Epicardial Adipose Tissue. Mediators Inflamm. 2019, 4075086. 10.1155/2019/4075086 30881222 PMC6383418

[B21] Olejarz W. Bryk D. Zapolska-Downar D. Malecki M. Stachurska A. Sitkiewicz D. (2014). Mycophenolic acid attenuates the tumour necrosis factor-alpha-mediated proinflammatory response in endothelial cells by blocking the MAPK/NF-kappaB and ROS pathways. Eur. J. Clin. Invest. 44, 54–64. 10.1111/eci.12191 24134657

[B22] Patil K. R. Mahajan U. B. Unger B. S. Goyal S. N. Belemkar S. Surana S. J. (2019). Animal Models of Inflammation for Screening of Anti-inflammatory Drugs: Implications for the Discovery and Development of Phytopharmaceuticals. Int. J. Mol. Sci. 20 (18), 4367. 10.3390/ijms20184367 PMC6770891 31491986

[B23] Sen Y. (2016). The Computational Pharmacology Study on A Traditional Chinese Medicine TaoRenHongHua Decoction for cardiovascular disease. A thesis submitted to Zhengzhou University for the degree of Master.

[B24] Sieve I. Ricke-Hoch M. Kasten M. Battmer K. Stapel B. Falk C. S. (2018). A positive feedback loop between IL-1beta, LPS and NEU1 may promote atherosclerosis by enhancing a pro-inflammatory state in monocytes and macrophages. Vascul. Pharmacol. 103–105, 16-28. 10.1016/j.vph.2018.01.005 29371126

[B25] Telinius N. Hjortdal Ve Dmsc P. (2019). Role of the lymphatic vasculature in cardiovascular medicine. Heart. 105 (23), 1777–1784. 10.1136/heartjnl-2018-314461 31585946

[B26] Tuttolomondo A. Di Raimondo D. Pecoraro R. Arnao V. Pinto A. Licata G. (2012). Atherosclerosis as an inflammatory disease. Curr. Pharm. Des. 18, 4266–4288. 10.2174/138161212802481237 22390643

[B27] Viatour P. Merville M. P. Bours V. Chariot A. (2005). Phosphorylation of NF-kappaB and IkappaB proteins: implications in cancer and inflammation. Trends Biochem. Sci. 30, 43–52. 10.1016/j.tibs.2004.11.009 15653325

[B28] Vuorio T. Tirronen A. Yla-Herttuala S. (2017). Cardiac Lymphatics - A New Avenue for Therapeutics? Trends Endocrinol. Metab. 28, 285–296. 10.1016/j.tem.2016.12.002 28087126

[B29] Wang Y. Cheng W. L. Wang Y. Peng J. P. Yuan J. Chen L. (2015). Qingre quyu granule stabilizes plaques through inhibiting the expression of tenascin-C in patients with severe carotid stenosis. Chin. J. Integr. Med. 21, 339–345. 10.1007/s11655-015-2161-y 25776840

[B30] Wei W. Xiao H. T. Bao W. R. Ma D. L. Leung C. H. Han X. Q. (2016). TLR-4 may mediate signaling pathways of Astragalus polysaccharide RAP induced cytokine expression of RAW264.7 cells. J. Ethnopharmacol. 179, 243–252. 10.1016/j.jep.2015.12.060 26743224

[B31] Wilhelmsen K. Xu F. Farrar K. Tran A. Khakpour S. Sundar S. (2015). Extracellular signal-regulated kinase 5 promotes acute cellular and systemic inflammation. Sci. Signal 8, ra86. 10.1126/scisignal.aaa3206 26307013 PMC5734625

[B32] Wu M. Zhang W. G. Liu L. T. (2017). Red yeast rice prevents atherosclerosis through regulating inflammatory signaling pathways. Chin. J. Integr. Med. 23, 689–695. 10.1007/s11655-017-2416-x 28861889

[B33] Yan J. Li J. Zhang L. Sun Y. Jiang J. Huang Y. (2018). Nrf2 protects against acute lung injury and inflammation by modulating TLR4 and Akt signaling. Free Radic. Biol. Med. 121, 78–85. 10.1016/j.freeradbiomed.2018.04.557 29678610

[B34] Yoshida N. Emoto T. Yamashita T. Watanabe H. Hayashi T. Tabata T. (2018). Bacteroides vulgatus and Bacteroides dorei Reduce Gut Microbial Lipopolysaccharide Production and Inhibit Atherosclerosis. Circulation 138, 2486–2498. 10.1161/CIRCULATIONAHA.118.033714 30571343

[B35] Yuan X. Li N. Zhang M. Lu C. Du Z. Zhu W. (2019). Taxifolin attenuates IMQ-induced murine psoriasis-like dermatitis by regulating T helper cell responses via Notch1 and JAK2/STAT3 signal pathways. BioMed. Pharmacother. 123, 109747. 10.1016/j.biopha.2019.109747 31881484

[B36] Zhang X. Xue C. Xu Q. Zhang Y. Li H. Li F. (2019). Caprylic acid suppresses inflammation via TLR4/NF-kappaB signaling and improves atherosclerosis in ApoE-deficient mice. Nutr. Metab. (Lond.) 16, 40. 10.1186/s12986-019-0359-2 31182969 PMC6555760

[B37] Weibin L. Zheng W. (2011). Taoren Honghua Jian Treatment of Sick Sinus Syndrome (Coronary Stenosis) 40 Cases. J. Pract. Tradit. Chin. Internal Med. 25, 48–49.

[B38] Zhou C. H. Liu L. Liu L. Zhang M. X. Guo H. Pan J. (2014). Salusin-beta not salusin-alpha promotes vascular inflammation in ApoE-deficient mice via the I-kappaBalpha/NF-kappaB pathway. PLoS One 9, e91468. 10.1371/journal.pone.0076714 24621517 PMC3951361

