# Supplementary material for: Taoren Honghua Drug Attenuates Atherosclerosis and Plays an Anti-Inflammatory Role in ApoE Knock-Out Mice and RAW264.7 Cells
Source: Front Pharmacol. 2020 Jul 17;11:1070. doi: 10.3389/fphar.2020.01070 (PMC7379336; doi:10.3389/fphar.2020.01070)
Supplement: Supplementary file 1 [file Presentation_1.pptx]

## Slide 1
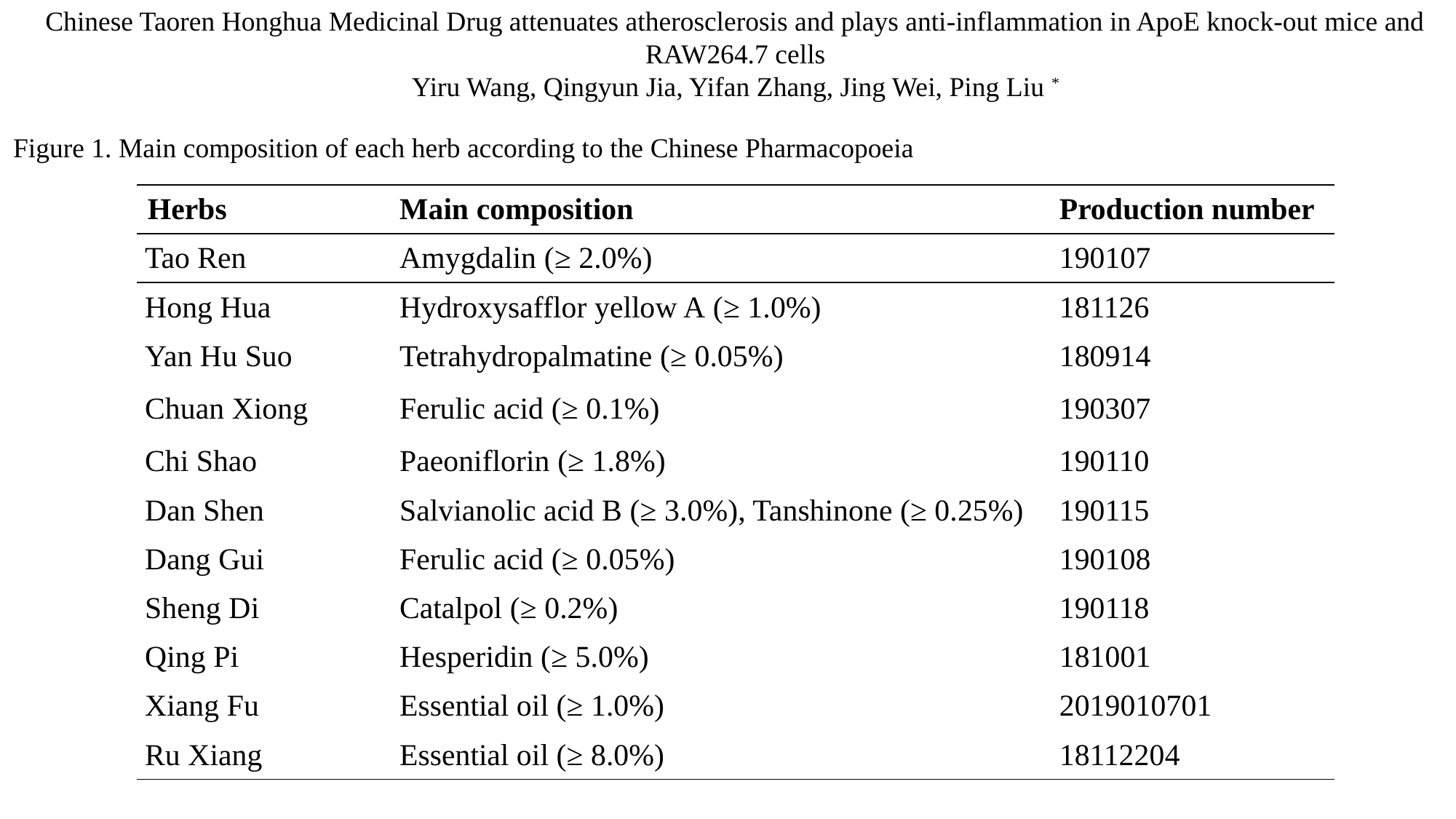

Chinese Taoren Honghua Medicinal Drug attenuates atherosclerosis and plays anti-inflammation in ApoE knock-out mice and RAW264.7 cells
Yiru Wang, Qingyun Jia, Yifan Zhang, Jing Wei, Ping Liu *
Figure 1. Main composition of each herb according to the Chinese Pharmacopoeia
| Herbs | Main composition | Production number |
| --- | --- | --- |
| Tao Ren | Amygdalin (≥ 2.0%) | 190107 |
| Hong Hua | Hydroxysafflor yellow A (≥ 1.0%) | 181126 |
| Yan Hu Suo | Tetrahydropalmatine (≥ 0.05%) | 180914 |
| Chuan Xiong | Ferulic acid (≥ 0.1%) | 190307 |
| Chi Shao | Paeoniflorin (≥ 1.8%) | 190110 |
| Dan Shen | Salvianolic acid B (≥ 3.0%), Tanshinone (≥ 0.25%) | 190115 |
| Dang Gui | Ferulic acid (≥ 0.05%) | 190108 |
| Sheng Di | Catalpol (≥ 0.2%) | 190118 |
| Qing Pi | Hesperidin (≥ 5.0%) | 181001 |
| Xiang Fu | Essential oil (≥ 1.0%) | 2019010701 |
| Ru Xiang | Essential oil (≥ 8.0%) | 18112204 |

## Slide 2
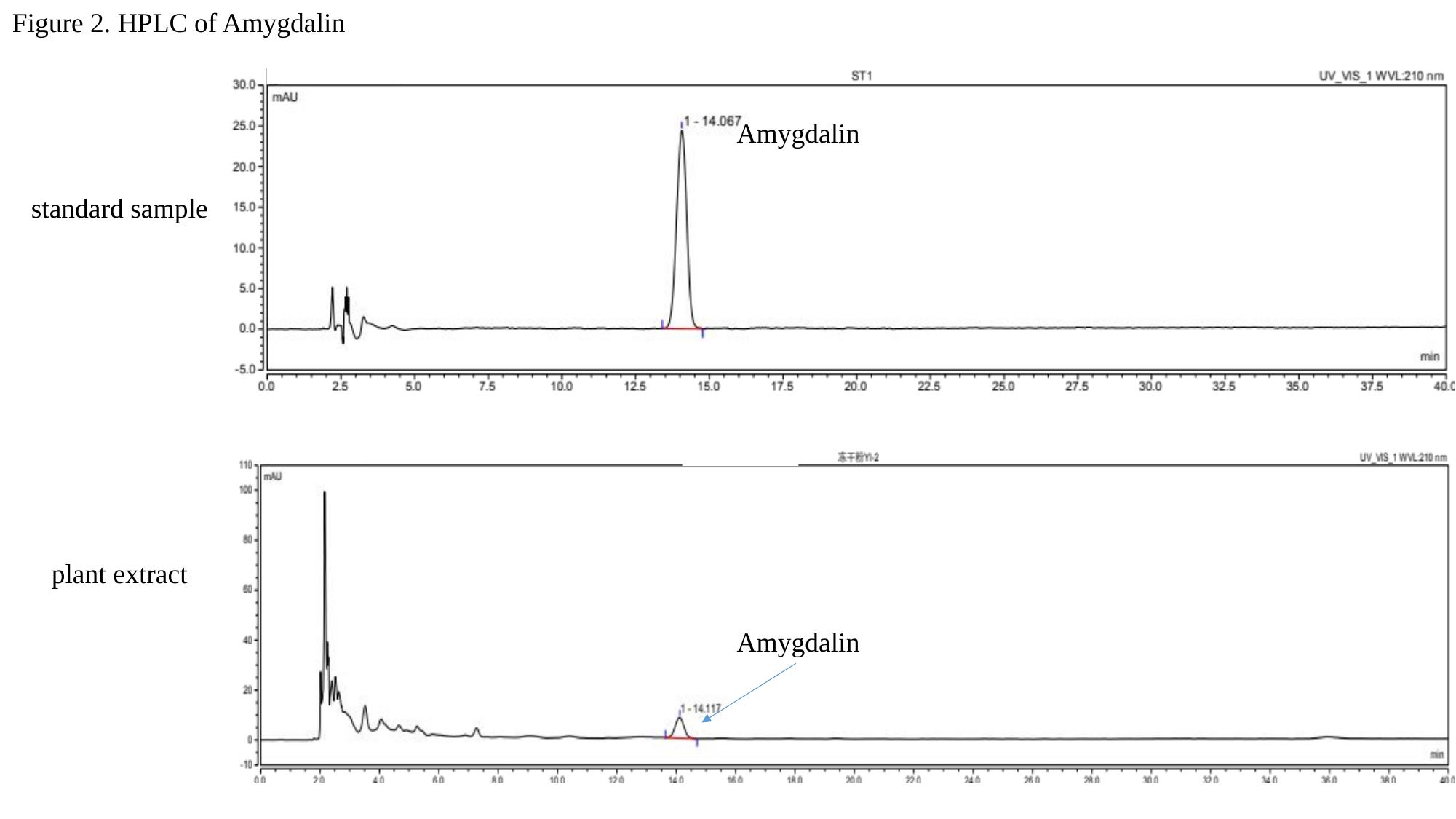

Figure 2. HPLC of Amygdalin
Amygdalin
standard sample
plant extract
Amygdalin

## Slide 3
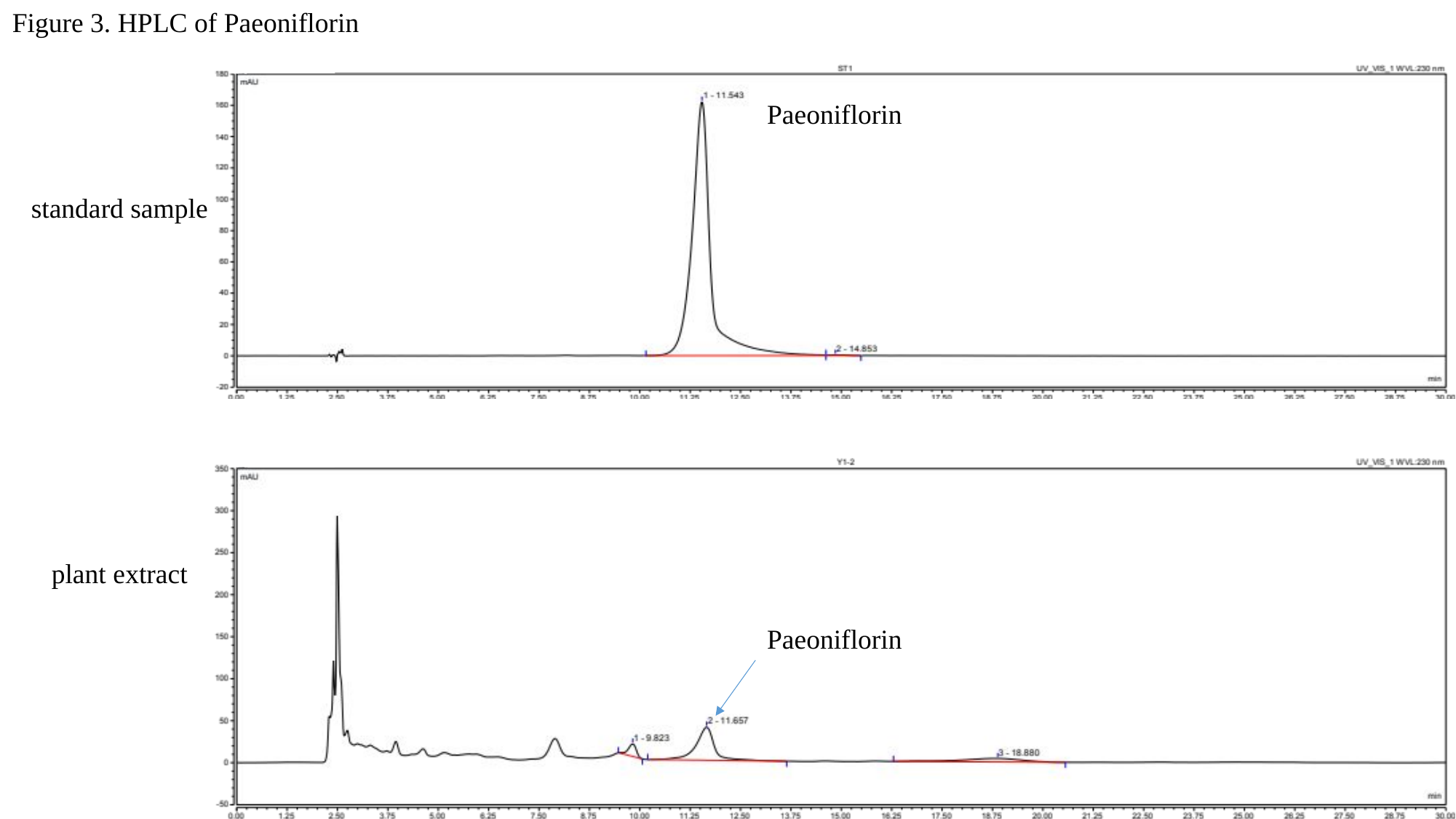

Figure 3. HPLC of Paeoniflorin
Paeoniflorin
standard sample
plant extract
Paeoniflorin

## Slide 4
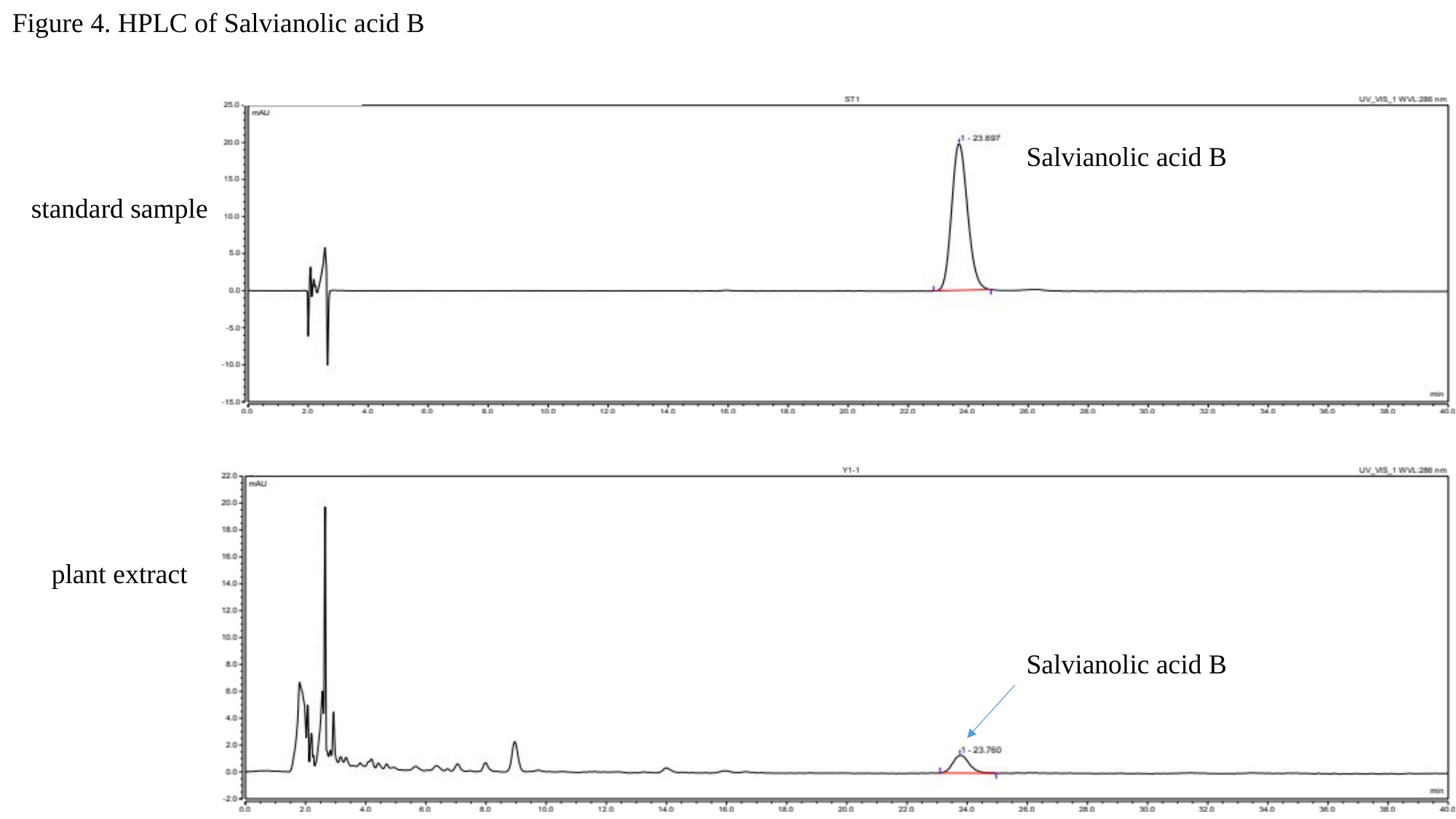

Figure 4. HPLC of Salvianolic acid B
Salvianolic acid B
standard sample
plant extract
Salvianolic acid B

## Slide 5
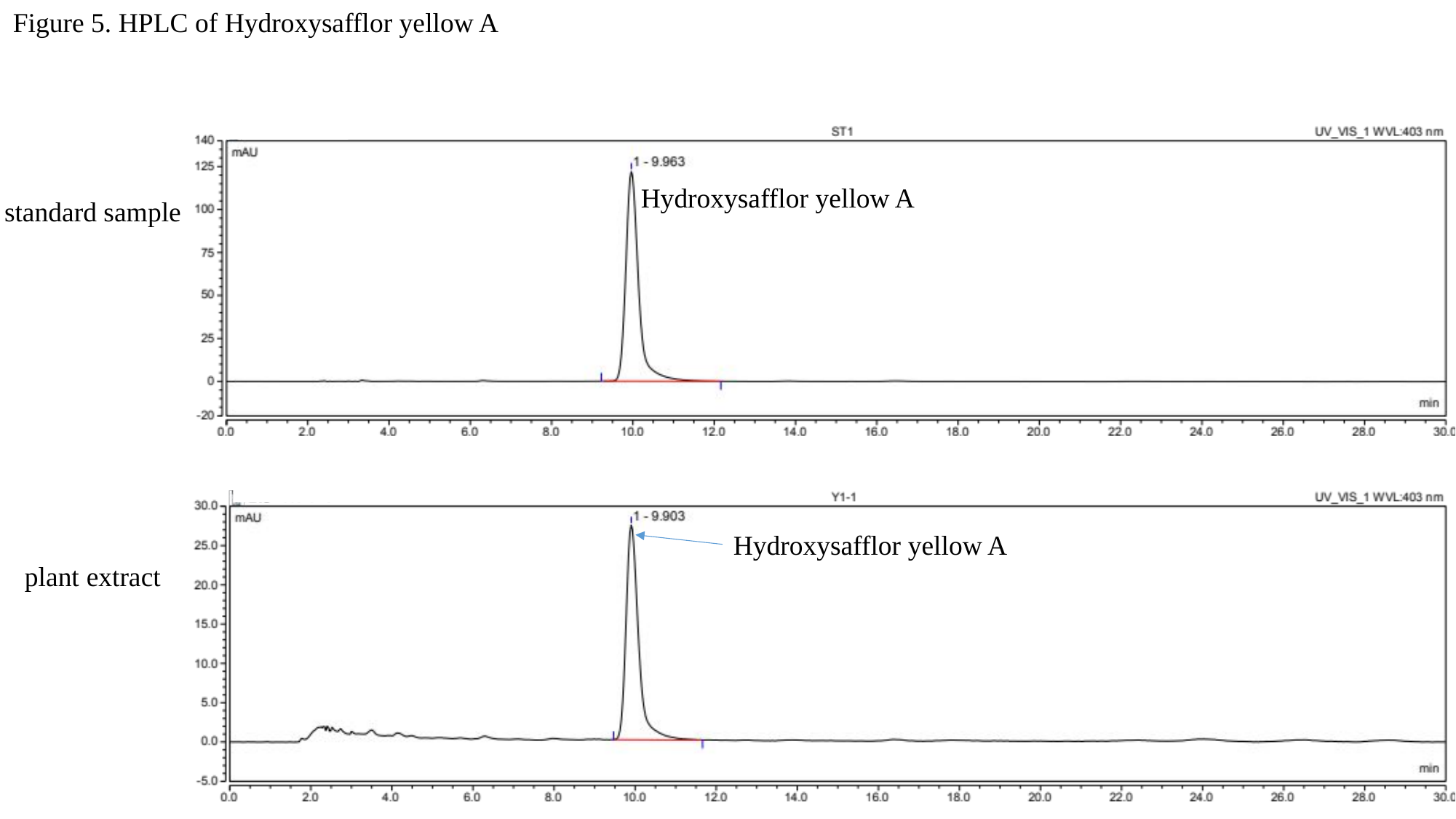

Figure 5. HPLC of Hydroxysafflor yellow A
Hydroxysafflor yellow A
standard sample
Hydroxysafflor yellow A
plant extract

## Slide 6
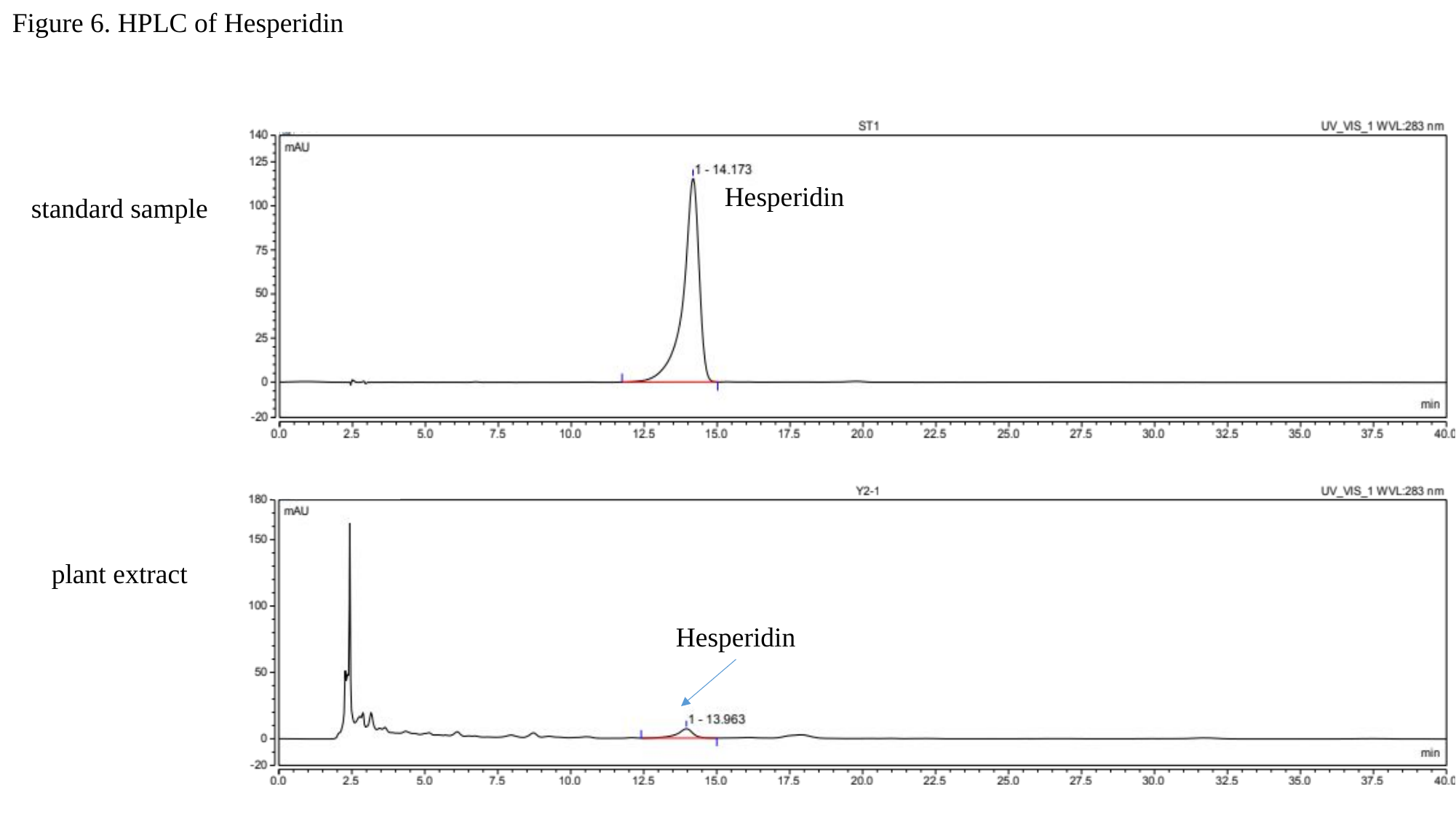

Figure 6. HPLC of Hesperidin
Hesperidin
standard sample
plant extract
Hesperidin

## Slide 7
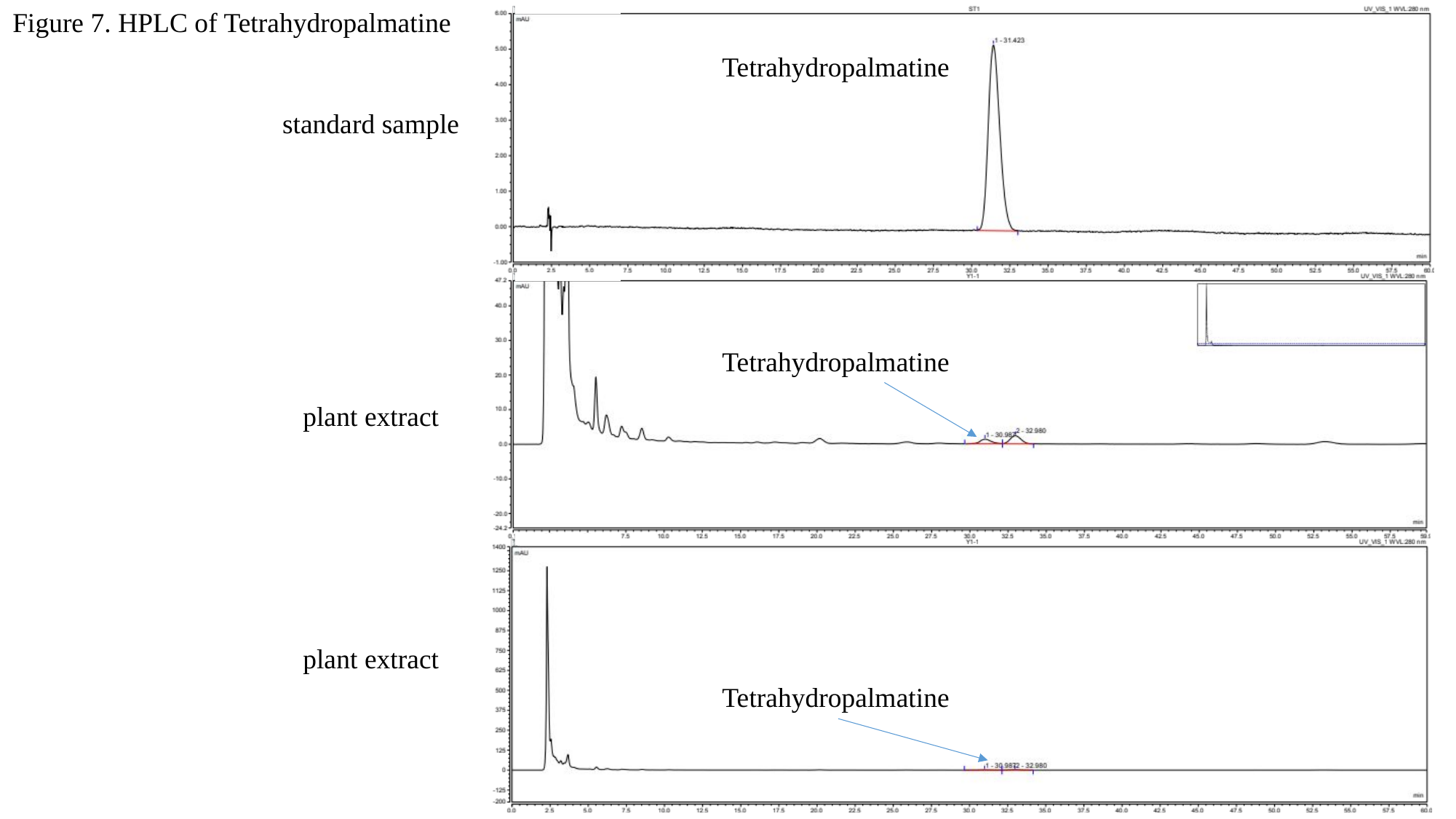

Figure 7. HPLC of Tetrahydropalmatine
Tetrahydropalmatine
standard sample
Tetrahydropalmatine
plant extract
plant extract
Tetrahydropalmatine

## Slide 8
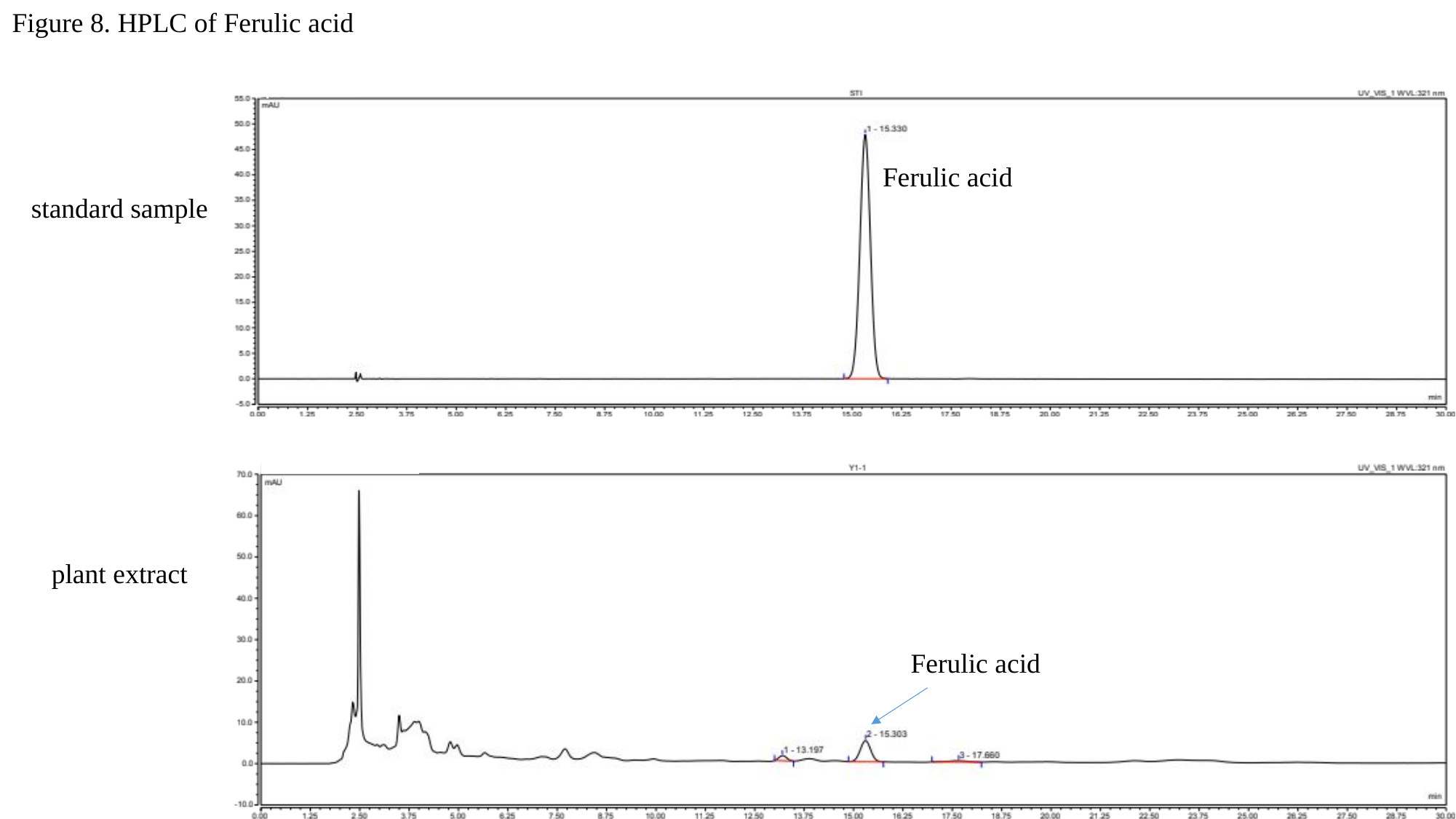

Figure 8. HPLC of Ferulic acid
Ferulic acid
standard sample
plant extract
Ferulic acid

## Slide 9
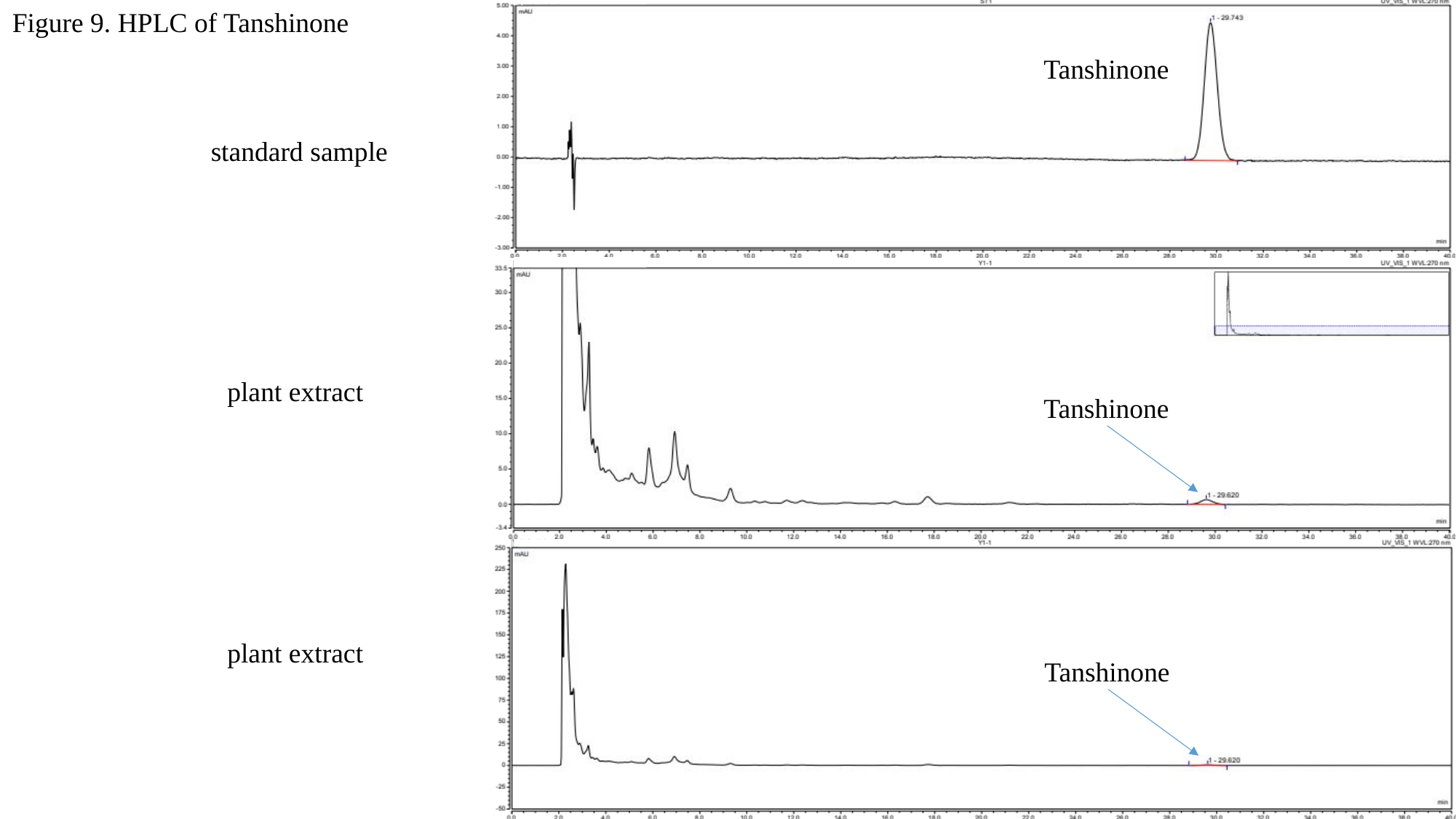

Figure 9. HPLC of Tanshinone
Tanshinone
standard sample
plant extract
Tanshinone
plant extract
Tanshinone

## Slide 10
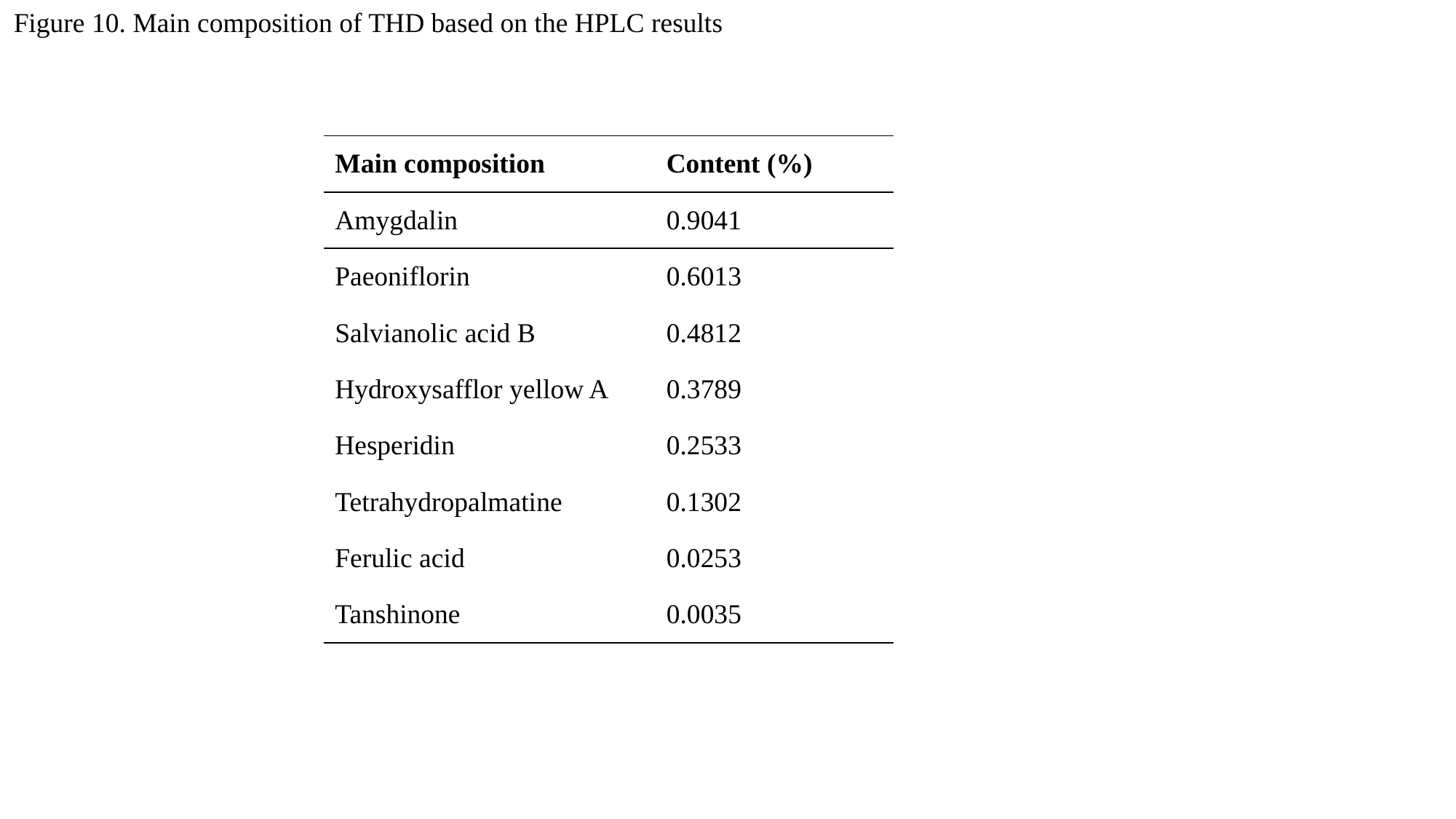

Figure 10. Main composition of THD based on the HPLC results
| Main composition | Content (%) |
| --- | --- |
| Amygdalin | 0.9041 |
| Paeoniflorin | 0.6013 |
| Salvianolic acid B | 0.4812 |
| Hydroxysafflor yellow A | 0.3789 |
| Hesperidin | 0.2533 |
| Tetrahydropalmatine | 0.1302 |
| Ferulic acid | 0.0253 |
| Tanshinone | 0.0035 |
